# Lack of Neuromuscular Fatigue in Singles Pickleball Tournament: A Preliminary Study

**DOI:** 10.3390/jfmk10030267

**Published:** 2025-07-16

**Authors:** Eric A. Martin, Steven B. Kim, George K. Beckham, James J. Annesi

**Affiliations:** 1 Kinesiology Department, Cal State Monterey Bay, Seaside, CA 93955, USA; gbeckham@csumb.edu (G.K.B.); jamesannesi@gmail.com (J.J.A.); 2 Department of Mathematics and Statistics, Cal State Monterey Bay, Seaside, CA 93955, USA; stkim@csumb.edu

**Keywords:** racquet sports, paddle sports, power, lower body force, countermovement jump, vertical jump

## Abstract

Objectives: The objective of this study was to examine the neuromuscular fatigue response to playing in a singles pickleball tournament, as measured by performance on a countermovement jump test (CMJ). We hypothesized that players would exhibit neuromuscular fatigue after the tournament. Methods: Six adult pickleball players (five male and one female, M ± SD: 40.2 ± 10.1 years old, height = 178.7 ± 12.3 cm, body mass = 85.4 ± 16.7 kg) participated in a 15 game singles pickleball tournament. Prior to the tournament, everyone completed the CMJ to assess lower body strength and power on paired Hawkin Dynamics force plates. After the tournament, players repeated the CMJ. Mixed-effects regression modeling was used to examine changes in key outcomes measured from the CMJ. Results: All nine outcomes from the CMJ significantly changed from pre to post-tournament (e.g., means for net impulse increased from 2.32 ± 0.22 to 2.40 ± 0.18 N·s, *p* = 0.0006; RSImod increased from 0.28 ± 0.07 to 0.33 ± 0.05, *p* = 0.0001, and propulsive peak power increased from 41.79 ± 6.14 to 44.34 ± 4.70 W/kg, *p* < 0.0001). All the changes demonstrated improved performance in the CMJ test. Seven out of the nine outcomes demonstrated a large effect size by the partial-eta square statistic, with η^2^-partial of 0.153–0.487, and three key outcomes (RSImod, propulsive peak power, and propulsive mean power) also demonstrated large effect sizes by the F^2^ statistic (F^2^ of 0.4603–0.9495). Conclusions: Contrary to our hypothesis, participants did not demonstrate significant neuromuscular fatigue. In contrast, they showed significant improvements in CMJ performance. It is possible that adequate rest between games prevented neuromuscular fatigue; alternately, singles pickleball may not provide enough stimulus in the lower body musculature to induce neuromuscular fatigue.

## 1. Introduction

Pickleball has been the fastest growing sport in the United States of America for three years in a row, according to the Sports & Fitness Industry Association [1,2]. Pickleball is a racquet/paddle sport played on a court that is 13.41 m long and 6.1 m wide. Like in tennis, the length of the court is divided in half by a net, with each player/doubles pair playing on their side and volleying the ball back and forth. Also like in tennis, pickleball can be played in either singles or doubles formats. Pickleball is considered a less physically demanding racquet/paddle sport compared to tennis, by virtue of the smaller court, shorter paddle (shorter lever length to generate forces), and use of a plastic ball with holes perforating it. The largest age group now playing pickleball is younger adults (25–34 years old), with the average age of what USA Pickleball dubs core players (those who play at least eight times per year) being 42 years old. The second largest demographic group is those aged 65 and older [1,2]. The increase in popularity, especially among older individuals, has led to a large increase in the absolute number of injuries related to pickleball [3,4]. However, there is little research on the physiological aspects of the sport, which would be a first step on the path to developing injury prevention strategies.

Most relevant prior physical research has focused on the basic cardiometabolic response to doubles pickleball [5,6,7,8], with only one study also including singles pickleball play [5]. This prior research has shown that pickleball, on average, provides a moderate intensity aerobic stimulus for middle age to older adults [5,7,8]. Webber et al. [5] stratified how much time during singles and doubles pickleball games is spent at light, moderate, and high intensity levels; they used both heart rate response and accelerometer data to quantify these levels. Based on average heart rate during play, a singles pickleball game can be stratified as 45.2% moderate and 27.5% each in light and vigorous intensities. However, according to accelerometer data, 80.5% of the time is spent at moderate intensity, 19.0% at light, and only 0.01% at a vigorous intensity. While doubles pickleball is of lower intensity [5], it has been shown to provide enough stimulus in middle age and older adults to trigger beneficial chronic adaptations in cardiovascular fitness and lower body muscular function [6,7]. However, it is not yet well studied how the stresses of playing either doubles or singles pickleball may contribute to fatigue. We published the only prior study on neuromuscular fatigue in pickleball [9]. Fatigue is an important topic to study in this area, because an increase in the fatigue one experiences through continued exercise increases the risk of suffering an injury [10,11].

In our work on this topic, we have used a variety of force-time derived characteristics of the countermovement jump test (CMJ) to evaluate the neuromuscular performance of the lower limbs. The force-time data derived from the CMJ are useful to pickleball, as higher levels of lower body force and power generalize to relevant tasks like movement around the court and transferring lower body force up the kinetic chain to contribute to stroke force. The CMJ has generally been considered valid and highly reliable for force production and assessing neuromuscular fatigue in a wide array of populations (e.g., general adult, athletes, elderly, people with chronic diseases) [12,13,14,15,16,17,18,19,20,21,22,23,24]. However, in our previous work in pickleball, we found a surprising result, i.e., that playing multiple games of recreational pickleball not only did not lead to neuromuscular fatigue on multiple force-time derived variables of the CMJ, but lead to a significant increase in force and power production [9,25]. This result was seen both in our first observational study, examining normal recreational play with uncontrolled rest periods and a variable number of games [9], as well as a pilot study follow-up that controlled the amount of play and rest times but with only four young males of high fitness levels [25]. In addition to the finding of no decreased performance in the CMJ across the games, we also observed a marked learning effect across baseline trials in our first study [9]. This was surprising, because most prior studies have demonstrated that the CMJ is reliable in general, athletic, and clinical populations, and they have not provided evidence of a learning effect [12,13,14,15,16,17,18,19,20,21,22,23,24]. This contrast is important to note, because if a learning effect exists across multiple trials, it may mask growing fatigue in the subjects (i.e., a participant may gain more performance from learning than they lose from fatigue). Both these sets of surprising results suggest that additional data are needed. Furthermore, all our prior work has been conducted in doubles pickleball; singles pickleball puts a greater stress on the body than doubles pickleball [5], as one player has to cover the same area of court as both players in doubles. Therefore, one would expect singles pickleball to cause fatigue.

The primary purpose of this study was to examine if singles pickleball would cause neuromuscular fatigue in the lower body in a general sample of male and female recreational pickleball players under controlled tournament conditions. We hypothesized that CMJ performance would decrease after playing a singles pickleball tournament. The secondary purpose was to examine inter-trial changes in CMJ performance, continuing the work of our previous research exploring potential learning effects in the CMJ test.

## 2. Materials and Methods

### 2.1. Participants

Six players (five males and one female) participated in a mixed-singles pickleball tournament. Inclusion criteria were adults with a pickleball rating of 2.5–3.5 by either self-rating or formal rating by any formal system, such as Dynamic Universal Pickleball Rating (DUPR). A DUPR rating of 2.5 represents the mid-point between novice and intermediate players, and 3.5 represents the midpoint between intermediate and advanced players [26]. This range was chosen to represent most recreational players who may be interested in tournament play. Exclusion criteria were having any injury at the time of the tournament which could make it unsafe for the participant to either perform the research tasks or play pickleball; injuries were screened for both at registration of interest and again when recruits showed up to the tournament (during the informed consent process). This study was approved by the Committee for the Protection of Human Subjects (protocol #270). All participants signed informed consent prior to participation in any research activities.

The six participants had a mean ± SD age of 40.2 ± 10.1 years (range: 25–52), height of 178.7 ± 12.3 cm, and body mass of 85.4 ± 16.7 kg. All participants regularly engaged in aerobic exercise, and all but one regularly engaged in resistance training. On average, players had only been playing pickleball for around two years, only played 1–2 times per week, and had only modest prior experience playing other racquet or paddle sports. At baseline, their CMJ performance was slightly below the average reported in young healthy adult populations [27,28] but much higher than the CMJ performance of middle age and older adult pickleball players [6]. In general, these players represented generally active people who were only recreational pickleball players. Due to the limited sample size, the outcome data were not analyzed by any of the demographic or training history factors.

### 2.2. Procedures

When participants arrived at the tournament site, after signing informed consent, they completed a demographic questionnaire to capture age and physical activity history, especially related to racquet/paddle sport experience. We measured their height using a Seca 213 stadiometer (Seca, Hamburg, Germany). Body mass was measured by the Hawkin Dynamics Force Plates (Gen 3, Hawkin Dynamics, Westbrook, ME, USA) during the quiet weighing period immediately prior to the CMJ test. Participants then performed the CMJ test (detailed below). After the CMJ test, participants were given approximately 10 min to warm up as they saw fit (both physical warm up exercises and hitting the ball) while the tournament brackets were constructed. Participants were called together, the tournament format was explained to them, then the tournament started (described in detail next). Immediately after the last game of the tournament, all participants repeated the CMJ test.

### 2.3. Tournament Format

The tournament format was a round-robin, where each participant played each other three times, for a total of 15 games; games were grouped into three sets of five, with small rest breaks given between games and larger rest breaks given between sets (detailed below). Each pair of opponents played simultaneously (i.e., three games occurred at the exact same time on three different courts).

Prior to the start of tournament play, all players were gathered together to review the tournament format and rules. Additionally, participants were instructed to play to the best of their ability at all times and to score as many points as possible, even if they were winning by a large margin. They were also instructed to not do anything to artificially slow down or speed up the normal play within the game, such as taking a long time to serve to run out the clock when they had a slight margin. Staff observed all games to keep score and saw no instances of any player engaging in any behavior that may have altered the pace of the game.

Each game lasted 8 min, starting and stopping at the blowing of the whistle by the researchers (if participants were in the middle of a rally when the stopping whistle blew, they were instructed to finish the rally and score the point if won). Participants received 5 min between games within a set to get water, find their next court assignment, and get to their next court. In between sets, participants were given 20 min of rest. This format of timed games, instead of the normal play-to-11 rules, was chosen to help ensure that all participants completed similar amounts of work and rest, in contrast to our first study where participants completed highly variable amounts of work and rest [9]. Throughout the tournament, players were allowed to eat and drink ad libitum.

### 2.4. CMJ Test

Pre-tournament, participants completed the CMJ test in pairs as they arrived and registered for the tournament; post-tournament, all participants lined up and completed each trial of the test one at a time. At each time point, when each person reached the front of the line, they completed the trial of the CMJ test, then returned to the back of the line. This format minimized between-participant variability in rest period between the end of the last game and CMJ testing. Pre-tournament, this led to rest periods of about 30 s between trials, while post-tournament, this led to a rest period of about 2 min between trials.

In the pre-tournament registration packet, a video describing and demonstrating the CMJ test was sent to participants to help familiarize them with the procedure. Furthermore, on the day, the researchers read from a standardized script of instructions, that included providing a live demonstration, to enhance reliability of the CMJ test.

All trials of the CMJ test were completed on paired Hawkin Dynamics Force Plates operating at 1000 Hz and analyzed using software from the manufacturer (Hawkin Capture App v9.5.0, Maths v17.1.0). Participants were instructed to complete the CMJs with their hands on their hips to eliminate the influence of arm swing [29]. The first four jumps were performed as warm up and familiarization: jumps 1–2 were at 50% of their maximal effort, and jumps 3–4 at 75% of their maximal effort. Then, jumps 5–8 were performed at full effort and recorded as the performance data. During the four maximum effort trials, researchers watched for incorrect jump mechanics (e.g., flexing the knees and holding them flexed during flight time, leading to a longer flight time than normal); if a participant performed a CMJ trial incorrectly, the researcher noted it on the data collection sheet and informed the participant not to do that again. No other coaching on jump technique was given.

To quantify different aspects of neuromuscular fatigue, a variety of measures were obtained from the CMJ either directly from the Hawkin Dynamics software (version 1.10) or with minor transformation via normalization to body mass in Microsoft Excel. It is worth noting that the Hawkin Dynamics software calculates jump height based on takeoff velocity. Previous studies have pointed out the necessity to consider how fatigue may affect primary markers of CMJ performance (e.g., jump height, peak power), as well as phase (e.g., braking, propulsion) and time characteristics [12,16,18,24]. To quantify aspects of the entire CMJ, the following dependent variables were obtained:

Overall performance variables:

- Jump height.
- Net impulse normalized to body mass (hereafter, net impulse).
- Time to takeoff.
- Reactive strength index modified (RSImod; jump height divided by time to takeoff).

Specific CMJ phase variables included:

- Braking phase mean power normalized to body mass (hereafter, braking mean power).
- Propulsive phase peak force normalized to body mass (hereafter, propulsive peak force).
- Propulsive phase peak power normalized to body mass (hereafter, propulsive peak power).
- Propulsive phase mean power normalized to body mass (hereafter, propulsive mean power).
- Propulsive phase time.

The CMJ performance variables selected were chosen to better understand how fatigue might manifest as changes in overall jumping ability (jump height, net impulse, RSImod, time to takeoff) and in the braking and propulsive phases of the CMJ. Measures of overall jumping performance are not always sensitive enough to reflect minor changes in fatigue, nor do they reflect why changes in CMJ performance may have occurred [12,16,18,24].

### 2.5. Statistical Analysis

To test whether CMJ performance changed throughout the study, we tested each dependent variable under the mixed-effects model using the lme4 version 1.1-10 package in R [30]. The mixed-effects model estimated the expected change of each of the ten dependent variables across all pre-tournament and post-tournament maximal effort jump trials (four trials at each time point; this was used as a fixed-effect), and the model accounted for repeated measures of each subject (random-effect). Individual trial data points (i.e., four trials for pre-tournament and four trials for post-tournament) were used for the purpose of hypothesis testing because utilizing the individual trial data points with the mixed-effects model is more powerful than utilizing the averaged data points with the standard paired t-test for comparing each variable between pre- and post-tournament. Under the mixed-effects model, the *j*th measurement of the *i*th subject (*y_ij_*) is modeled as follows: *y_ij_* = β_0*i*_ + β_1_ *x_ij_* + ε*_ij_* where *x_ij_* is a dummy-coded variable representing the time point (*x_ij_* = 1 for the post-tournament measurement and *x_ij_* = 0 for the pre-tournament measurement), β_0*i*_ accounts for between-subject variability (random-effect), β_1_ quantifies the change in the expected outcome between the pre- and post-tournament (fixed-effect), and ε*_ij_* is the random error. We used the *T* test for β_1_ under the mixed-effects model, calculated T statistics and *p*-values (*T* test for β_1_) using the lmerTest package version 2.0-36 in R [31], and calculated F^2^ and η^2^-partial to quantify the effect sizes using the cohens_f_squared and t_to_eta2 functions within the effectsize package version 1.0.1 in R [32]. The F^2^ is defined as [(R_1_)^2^ − (R_0_)^2^]/[1 − (R_1_)^2^] where (R_1_)^2^ is the R-squared value of the full model which includes β_1_ in the mixed-effects model and (R_0_)^2^ is the R-square value of the reduced (null) model which assumes β_1_ = 0 in the model. The η^2^-partial is calculated by SS_T_/(SS_T_ + SS_E_) where SS_T_ is the sum of squares due to the treatment (pre- or post-tournament) and SS_E_ is the sum of squares unexplained under the model. Per standard interpretation, F^2^ values of <0.02 are considered trivial, ≥0.02 but <0.15 is small, ≥0.15 but <0.35 is medium, and ≥0.35 is large; η^2^-partial values were interpreted as <0.01 is trivial, ≥0.01 but <0.06 is small, ≥0.06 but <0.14 is medium, and ≥0.14 is large [33,34]. The intraclass correlation coefficient (ICC), defined as V(β_0*i*_)/[V(β_0*i*_) + V(ε*_i_*)], was estimated for pre- and post-tournament measurement using the icc function within the performance package version 0.15.0 in R [35]. The normality assumption was assessed by the Q-Q plot and Shapiro-Wilk test.

To examine potential learning effects, four inter-trial comparisons were done for each CMJ performance variable. One-way repeated-measure ANOVA was performed separately for the pre-tournament and post-tournament jumps. The post-hoc tests were performed using the Bonferroni correction particularly for these comparisons: (1) pre-tournament Trial 2 vs. Trial 1, (2) pre-tournament Trial 4 vs. Trial 1, (3) post-tournament Trial 2 vs. Trial 1, and (4) post-tournament Trial 4 vs. Trial 1. For the analysis of each inter-trial comparison, the *p*-value was adjusted using the Bonferroni correction, and the Cohen’s d was calculated.

## 3. Results

Performance on the outcomes from the CMJ test are presented in Table 1. On average, the group of participants significantly improved their jump performance on all metrics, with medium to large effect sizes except for breaking mean power and propulsive peak force according to F^2^. Participants lost body mass on average, and this was found to be statistically significant despite the small sample size, however the average amount of body mass loss is likely not clinically relevant, is likely due to loss of water weight from sweating, and would be expected after hours of physical activity. For the jump height, net impulse, time to takeoff, and propulsive peak power, one or two data points affected the normality assumption. We re-fitted the model without those data points, and we confirmed that they did not affect the conclusion.

With the aim of ascertaining if there was an improvement across trials within the pre- or post-testing period on any of the CMJ outcomes, a one-way repeated-measure ANOVA was done on each outcome separately for the pre-tournament and post-tournament jumps. None of the CMJ outcomes were significantly different between trials for the pre-tournament and post-tournament jumps (all *p*-values were greater than 0.05). The Cohen’s d effect size indicated potential differences between the first and fourth pre-tournament trial on a few key outcomes: jump height, net impulse, and propulsive peak power. However, with such a small sample size, no *p* value (using the Bonferroni correction) reached statistical significance as reported in Table 2.

## 4. Discussion

Contrary to our hypothesis, we did not see signs of neuromuscular fatigue in the CMJ after a singles pickleball tournament. While our prior work had not shown signs of neuromuscular fatigue in doubles pickleball [9,25], we had anticipated that the greater workload and work rate inherent to singles play [5] and large total length of play in the present study (15 games for a total of 120 min of playing versus 9 games or less in our previous work [9,25]) would be sufficient to induce reduced performance in the CMJ. One possible explanation is that the amount of rest given in the current tournament (5 min between matches, 20 min between sets of games, for a total 120 min of work and 100 min rest) was enough to prevent significant neuromuscular fatigue. Even during a match, there is non-active time while one player retrieves a ball, sets up for a serve, etc., effectively increasing the rest within games. Additionally, moderate intensity exercise makes up the majority of the game (measured by accelerometry: 80.5 ± 13.3% moderate intensity, 19.0 ± 13.5% light intensity, and 0.01 ± 0.02% vigorous intensity [5]). The very low proportion of vigorous exercise classified may also indicate that singles pickleball does not provide enough lower body loading to induce neuromuscular fatigue. Future studies will need to directly measure the amount or load of pickleball play while examining markers of fatigue to connect the work done in-game to changes in performance.

The lack of fatigue, and instead an increase in CMJ performance, seen in this study is similar to what has been seen among high-level padel players [36]. Carnero-Diaz et al. [36] found that the top Andalusian padel players experienced a significant increase in CMJ height of 2.5 cm (8.42% increase above baseline, *p* = 0.006), which is similar to the present study. The increase in CMJ height happened whether the padel match consisted of two or three sets, with no significant difference between sets. They also assessed neuromuscular performance of the upper limbs using handgrip dynamometery and found similar improvements in grip strength over time. While Carnero-Diaz et al. [36] did not include length of game play in their study, a literature review of studies reporting activity profiles of padel game play indicate that padel games lasted 17–30 min on average (longer than in the present study), with players having 1.28–2.51 times more rest than effective work time during a game [37]. To date, no studies describe the lengths of work and rest during pickleball games, however a very recent pair of publications indicated that 86.6–87.3% of rallies in professional singles pickleball games were short or medium duration (defined overall as eight hits or less) [38,39]; this implies the active periods of play are very short time wise, with frequent periods of inactivity for ball retrieval, etc. Furthermore, reported heart rates during singles and doubles pickleball [5,7] are lower than those reported in padel [37], indicating a generally lower exercise intensity in pickleball. The lack of fatigue seen in the present study and in padel [36] is in contrast to what Armstrong et al. [40] observed after a 60 min continuous padel game simulation. Therefore, it may be possible that either padel or pickleball could induce neuromuscular fatigue if a sufficient amount of play without rest is performed, albeit to an extent that may not reflect real world play of the sport under its normal rules.

Fatigue is a multi-dimensional concept, and neuromuscular fatigue is only one way to examine it. While not captured empirically in this study, at the end of the tournament, participants reported feeling tired, perhaps indicating that they experienced subjective fatigue. Carnero-Diaz et al. [36] assessed subjective fatigue due to padel games using the category ratio (CR-10) rating of perceived exertion (RPE) scale. While they found a significant correlation between game duration and RPE (*p* < 0.001, *r* = 0.574), there was not a significant correlation between RPE and either CMJ change or handgrip performance change. These results indicate a lack of relationship between subjective fatigue and fatigue effects on neuromuscular performance. In our study, we did not formally capture subjective feelings of fatigue. However, after the singles pickleball tournament, participants described feeling fatigued, despite their improved CMJ performance. In future studies, we plan to use a questionnaire to capture subjective feelings of fatigue, to see if pickleball can cause fatigue in other domains besides lower body neuromuscular performance.

In our prior research, we found a significant learning effect across trials within the pre-pickleball game set of jumps [9]. The data from the present study did not demonstrate a similar learning effect. The probable reasons were that the current sample was younger on average (mean age of 40 instead of 60); engaged in more regular exercise, especially resistance training; and jumped higher (27 cm compared to 13 cm), i.e., had objectively higher levels of fitness. Besides these contributions, the small sample size may have limited our ability to detect a learning effect if one was present. Prior research has generally not demonstrated a significant learning effect in vertical jump testing [14,19,21,22,23]; however, many of those studies were done with more experienced athletes or with less sensitive equipment and measures (i.e., jump mats and only calculating jump height from flight time). The learning effect and reliability of baseline CMJ testing in recreational pickleball players still warrant further investigation. Furthermore, discerning how much learning effect contributed to the increase from pre- to post-tournament in CMJ performance needs further investigation.

Adequate rest alone may explain a lack of fatigue but does not explain the significant increases in CMJ performance seen in this study. Though we did not detect an acute learning effect within the pre-tournament trials in the present study, it is possible that learning effect contributed to the post-tournament increase. The expected enhancements in CMJ performance from warm up (increased muscle temperature and associated enhanced activation; [41,42]), and possibly some form of post-activation potentiation [43,44] could also be contributing factors. In the current study design, with only two measurement time points, we were unable to detect more than a linear increase. More frequent sampling throughout the tournament may allow detection of other possible trends (e.g., logarithmic, where improvements level off over time; or parabolic, in which improvements peak during in the tournament then taper off due to fatigue).

The practical application from this study is for players and tournament organizers to help determine appropriate rest times. Recreational players, who may play multiple games in a chosen session, can feel confident that a short break between each match, for up to five games, interspersed with periodic moderate breaks of 20 min, can help them maintain their lower body power. These guidelines can also help tournament organizers balance play and rest times, though they should recognize that the current study only used 8 min of play, which may or may not represent normal game length tournament play, especially in tournaments that play to 15 points instead of 11.

The primary limitation of this study was the small sample size; due to lack of sign ups and no-shows to the scheduled tournament, we ended up with a sample size one quarter of what we designed the procedures for. However, despite the small sample size, the statistical power was still strong, as evidenced by most *p* values for change in CMJ parameters over time being <0.01, and most of the accompanying effect sizes being large. One of the strengths of the current study was strict control of play and rest time to help ensure all players experienced a similar workload. However, there was a small difference in the amount of inter-trial rest on the CMJ between the pre-tournament and post-tournament periods. Pre-tournament, players were tested in pairs as they registered to help move the whole process along. Post-tournament, all players performed the test as one group. The differences in inter-trial rest could have theoretically impacted the inter-trial difference comparison at post-test compared to pre-test, however since we found no significant differences between CMJ trials, we do not have evidence that the differential inter-trial rest periods impacted the study results. More importantly, the different rest periods likely have a negligible impact on the main finding, that there was not a fatigue effect from the tournament overall. In future studies, we will more strictly control for the rest period. Future research could also control for water and food intake, as in the present study people were allowed to eat and drink ad libitum. While the present sample was representative in age and fitness of the general population who usually plays singles pickleball, it may not be generalizable to older pickleball players if they chose to play singles. Furthermore, with only one female participant, these results may not generalize to a larger female population.

## 5. Conclusions

Playing in a 15 game singles pickleball tournament, with controlled game times and adequate rest, does not induce neuromuscular fatigue in the lower body compared to baseline levels in young to middle-aged adult recreational pickleball players.

## Figures and Tables

**Table 1:** Changes in Measures of CMJ Performance.

Outcome	Pre-Tournament Mean (SD) [Range]	Post-Tournament Mean (SD) [Range]	Change (β_1_)	*p* Value	*F* ^2^	η^2^-Partial	Pre-Tournament ICC	Post-Tournament ICC
Body mass (kg)	85.19 (18.60) [58.01–106.76]	84.92 (18.30) [57.81–106.84]	−0.28 (0.86) [−1.53 to 0.56]	**0.0471**	0.1022	0.0927 *	0.9999	1.0000
Jump height (cm)	27.33 (5.09) [20.95–34.17]	29.11 (4.25) [22.73–34.70]	1.79 (1.77) [−1.72 to 3.10]	**0.0008**	0.3187 *	0.2417 **	0.8678	0.9274
Net impulse (N*s)	2.32 (0.22) [2.04–2.60]	2.40 (0.18) [2.13–2.63]	0.08 (0.07) [−0.07 to0.14]	**0.0006**	0.3413 *	0.2545 **	0.8553	0.9330
Time to takeoff (s)	0.98 (0.12) [0.82–1.08]	0.90 (0.14) [0.78–1.13]	−0.08 (0.13) [−0.28 to 0.06]	**0.0058**	0.2068 *	0.1714 **	0.5462	0.8536
RSImod	0.28 (0.07) [0.21–0.40]	0.33 (0.05) [0.28–0.41]	0.04 (0.05) [−0.03 to 0.12]	**0.0001**	0.4603 **	0.3152 **	0.8230	0.7643
Braking mean power (W/kg)	−9.33 (2.78) [−13.47 to −5.41]	−10.08 (2.75) [−13.98 to −6.31]	−0.75 (0.99) [−2.41 to 0.64]	**0.0183**	0.1472	0.1283 *	0.7720	0.8651
Propulsive peak force (N/kg)	214.40 (9.52) [198.73–225.29]	218.93 (10.27) [210.41–236.32]	4.53 (7.26) [−6.19 to 11.68]	**0.0400**	0.1098	0.0989 *	0.4022	0.6223
Propulsive peak power (W/kg)	41.79 (6.14) [34.78–50.38]	44.34 (4.70) [38.09–49.78]	2.55 (2.01) [−1.37 to 4.20]	**<0.0001**	0.9495 **	0.4870 **	0.9452	0.9737
Propulsive mean power (W/kg)	22.83 (2.87) [19.19–26.40]	24.22 (2.40) [20.88–27.93]	1.39 (1.55) [−1.30 to 3.44]	**0.0001**	0.4725 **	0.3209 **	0.8519	0.9023
Propulsive phase time (s)	0.30 (0.02) [0.29–0.34]	0.29 (0.01) [0.27–0.31]	−0.02 (0.03) [−0.07 to 0.01]	**0.0096**	0.1800 *	0.1525 **	0.5027	0.4464

Notes: bold test indicates significant *p* values, * = medium effect size, ** = large effect size.

**Table 2:** Inter-trial comparisons (difference) of jump performance.

		Pre-Tournament Trials 2 and 1	Pre-Tournament Trials 4 and 1	Post-Tournament Trials 2 and 1	Post-Tournament Trials 4 and 1
Jump height (cm)	Mean (SD)	−0.32 (1.43)	−1.07 (2.00)	−0.34 (1.28)	0.36 (1.54)
	*p* value	1.0000	0.4966	1.0000	1.0000
	d	−0.2219	−0.5334 *	−0.2664	0.2336
Net impulse (N*s)	Mean (SD)	−0.01 (0.06)	−0.05 (0.08)	−0.02 (0.06)	0.01 (0.07)
	*p* value	1.0000	0.4530	0.9840	1.0000
	d	−0.2313	−0.5629 *	−0.3026	0.1928
Time to takeoff (s)	Mean (SD)	−0.00 (0.09)	0.03 (0.20)	−0.05 (0.09)	−0.01 (0.08)
	*p* value	1.0000	1.0000	0.4644	1.0000
	d	−0.0479	0.1281	−0.5549 *	−0.1607
RSImod	Mean (SD)	−0.01 (0.04)	−0.02 (0.06)	0.01 (0.03)	0.01 (0.04)
	*p* value	1.0000	0.6804	0.6886	1.0000
	d	−0.1735	−0.4302	0.4262	0.2748
Braking mean power (W)	Mean (SD)	−0.50 (2.37)	−0.38 (2.67)	−0.94 (1.33)	−0.81 (1.65)
	*p* value	1.0000	1.0000	0.2900	0.5706
	d	−0.2113	−0.1434	−0.7045 *	−0.4883
Propulsive peak force (N)	Mean (SD)	2.96 (17.35)	−2.68 (18.70)	6.30 (8.16)	5.91 (9.25)
	*p* value	1.0000	1.0000	0.2350	0.3566
	d	0.1706	−0.1432	0.7713 *	0.6390 *
Propulsive peak power	Mean (SD)	−0.48 (1.66)	−1.11 (1.70)	−0.53 (0.97)	−0.70 (1.40)
	*p* value	1.0000	0.3444	0.4704	0.5520
	d	−0.2917	−0.6501 *	−0.5507 *	−0.4991
Propulsive mean power (W)	Mean (SD)	−0.22 (1.87)	−0.90 (2.03)	0.12 (1.02)	0.14 (0.58)
	*p* value	1.0000	0.6568	1.0000	1.0000
	d	−0.1175	−0.4419	0.1223	0.2451
Propulsive phase time (s)	Mean (SD)	0.00 (0.02)	0.01 (0.04)	−0.01 (0.03)	0.00 (0.02)
	*p* value	1.0000	0.9308	1.0000	1.0000
	d	0.1397	0.3224	−0.2943	0.1419

Notes: mean is the difference between the two compared trials; * = medium effect size.

## Data Availability

Data are publicly archived at https://osf.io/vfja5/ (accessed on 8 July 2025).

## References

[1] Sports & Fitness Industry Association (2024). Sports, Fitness, and Leisure Activities Topline Participation Report.

[2] Sports & Fitness Industry Association, Pickleheads (2024). State of Pickleball: Participation & Infrastructure Report. https://www.sportstravelmagazine.com/sfia-and-pickleheads-release-participation-infrastructure-report/.

[3] Forrester M.B. (2020). Pickleball-Related Injuries Treated in Emergency Departments. J. Emerg. Med..

[4] Greiner N. (2019). Pickleball: Injury Considerations in an Increasingly Popular Sport. Mo. Med..

[5] Webber S.C., Anderson S., Biccum L., Jin S., Khawashki S., Tittlemier B.J. (2023). Physical Activity Intensity of Singles and Doubles Pickleball in Older Adults. J. Aging Phys. Act..

[6] Wray P., Ward C.K., Nelson C., Sulzer S.H., Dakin C.J., Thompson B.J., Vierimaa M., Das Gupta D., Bolton D.A.E. (2021). Pickleball for Inactive Mid-Life and Older Adults in Rural Utah: A Feasibility Study. Int. J. Environ. Res. Public Health.

[7] Smith L.E., Buchanan C.A., Dalleck L.C. (2018). The Acute and Chronic Physiological Responses to Pickleball in Middle-Aged and Older Adults. Int. J. Res. Exerc. Physiol..

[8] Denning W.M., Zagrodnik J., Smith M., Ruden T. (2022). Physical Activity Differences between Walking and Playing Pickleball Doubles. Sci. Sports.

[9] Martin E., Ritchey M., Kim S., Falknor M., Beckham G. (2024). Lack of Neuromuscular Fatigue Due to Recreational Doubles Pickleball. Multidiscip. Sci. J..

[10] Jones C.M., Griffiths P.C., Mellalieu S.D. (2017). Training Load and Fatigue Marker Associations with Injury and Illness: A Systematic Review of Longitudinal Studies. Sports Med..

[11] Chappell J.D., Herman D.C., Knight B.S., Kirkendall D.T., Garrett W.E., Yu B. (2005). Effect of Fatigue on Knee Kinetics and Kinematics in Stop-Jump Tasks. Am. J. Sports Med..

[12] Claudino J.G., Cronin J., Mezêncio B., McMaster D.T., McGuigan M., Tricoli V., Amadio A.C., Serrão J.C. (2017). The Countermovement Jump to Monitor Neuromuscular Status: A meta-analysis. J. Sci. Med. Sport..

[13] Singh H., Kim D., Kim E., Bemben M.G., Anderson M., Seo D.I., Bemben D.A. (2014). Jump Test Performance and Sarcopenia Status in Men and Women, 55 to 75 years of age. J. Geriatr. Phys. Ther..

[14] Buehring B., Krueger D., Fidler E., Gangnon R., Heiderscheit B., Binkley N. (2015). Reproducibility of Jumping Mechanography and Traditional Measures of Physical and Muscle Function in Older Adults. Osteoporos. Int..

[15] Parsons C.M., Edwards M.H., Cooper C., Dennison E.M., Ward K.A. (2020). Are Jumping Mechanography Assessed Muscle Force and Power, and Traditional Physical Capability Measures Associated with Falls in Older Adults? Results from the Hertfordshire Cohort Study. J. Musculoskelet. Neuronal Interact..

[16] Watkins C.M., Barillas S.R., Wong M.A., Archer D.C., Dobbs I.J., Lockie R.G., Coburn J.W., Tran T.T., Brown L.E. (2017). Determination of Vertical Jump as a Measure of Neuromuscular Readiness and Fatigue. J. Strength. Cond. Res..

[17] Watkins C.M., Gill N.D., McGuigan M.R., Maunder E., Spence A.J., Downes P., Neville J., Storey A.G. (2024). Kinetic Analysis, Potentiation, and Fatigue During Vertical and Horizontal Plyometric Training: An In-Depth Investigation Into Session Volume. Int. J. Sports Physiol. Perform..

[18] Gathercole R., Sporer B., Stellingwerff T., Sleivert G. (2015). Alternative Countermovement-Jump Analysis to Quantify Acute Neuromuscular Fatigue. Int. J. Sports Physiol. Perform..

[19] Gathercole R.J., Stellingwerff T., Sporer B.C. (2015). Effect of Acute Fatigue and Training Adaptation on Countermovement Jump Performance in Elite Snowboard Cross Athletes. J. Strength. Cond. Res..

[20] Santos C.A.F., Amirato G.R., Jacinto A.F., Pedrosa A.V., Caldo-Silva A., Sampaio A.R., Pimenta N., Santos J.M.B., Pochini A., Bachi A.L.L. (2022). Vertical Jump Tests: A Safe Instrument to Improve the Accuracy of the Functional Capacity Assessment in Robust Older Women. Healthcare.

[21] Bellicha A., Giroux C., Ciangura C., Menoux D., Thoumie P., Oppert J.M., Portero P. (2022). Vertical Jump on a Force Plate for Assessing Muscle Strength and Power in Women with Severe Obesity: Reliability, Validity, and Relations with Body Composition. J. Strength. Cond. Res..

[22] Manor J., Bunn J., Bohannon R.W. (2020). Validity and Reliability of Jump Height Measurements Obtained From Nonathletic Populations with the VERT Device. J. Geriatr. Phys. Ther..

[23] Farias D.L., Teixeira T.G., Madrid B., Pinho D., Boullosa D.A., Prestes J. (2013). Reliability of Vertical Jump Performance Evaluated with Contact Mat in Elderly Women. Clin. Physiol. Funct. Imaging.

[24] Bishop C., Jordan M., Torres-Ronda L., Loturco I., Harry J., Virgile A., Mundy P., Turner A., Comfort P. (2023). Selecting Metrics That Matter: Comparing the Use of the Countermovement Jump for Performance Profiling, Neuromuscular Fatigue Monitoring, and Injury Rehabilitation Testing. Strength. Cond. J..

[25] Ritchey M., Falknor M., Beckham G., Martin E. (2024). Pilot Study of Neuromuscular Fatigue After Doubles Pickleball Tournament. Int. J. Exerc. Sci. Conf. Proc..

[26] DUPR INC How It Works. https://www.dupr.com/how-it-works.

[27] Markovic G., Dizdar D., Jukic I., Cardinale M. (2004). Reliability and Factorial Validity of Squat and Countermovement Jump Tests. J. Strength. Cond. Res..

[28] McKay M.J., Baldwin J.N., Ferreira P., Simic M., Vanicek N., Burns J. (2017). Reference Values for Developing Responsive Functional Outcome Measures across the Lifespan. Neurology.

[29] Lees A., Vanrenterghem J., Clercq D.D. (2004). Understanding How an Arm Swing Enhances Performance in the Vertical Jump. J. Biomech..

[30] Bates D., Mächler M., Bolker B., Walker S. (2015). Fitting Linear Mixed-Effects Models Using lme4. J. Stat. Softw..

[31] Kuznetsova A., Brockhoff P.B., Christensen R.H.B. (2017). lmerTest Package: Tests in Linear Mixed Effects Models. J. Stat. Softw..

[32] Ben-Shachar M.S., Lüdecke D., Makowski D. (2020). effectsize: Estimation of Effect Size Indices and Standardized Parameters. J. Open Source Softw..

[33] Cohen J. (2013). Statistical Power Analysis for the Behavioral Sciences.

[34] Bland J.M., Altman D.G. (1986). Statistical Methods for Assessing Agreement between Two Methods of Clinical Measurement. Lancet.

[35] Lüdecke D., Ben-Shachar M.S., Patil I., Waggoner P., Dominique M. (2021). Performance: An R Package for Assessment, Comparison and Testing of Statistical Models. J. Open Source Softw..

[36] Carnero-Diaz Á., Nuñez-González J.L., Fernández D.E.O.-F.A.I., Pecci J. (2024). Influence of Competitive Padel Matches on Physical Fitness and Perceptual Responses in High-Level Players. J. Sports Med. Phys. Fit..

[37] Mellado-Arbelo O., Baiget E. (2022). Activity Profile and Physiological Demand of Padel Match Play: A Systematic Review. Kinesiology.

[38] Prieto-Lage I., Reguera-López-de-la-Osa X., Juncal-López A., Silva-Pinto A.J., Argibay-González J.C., Gutiérrez-Santiago A. (2024). Notational Analysis of Men’s Singles Pickleball: Game Patterns and Competitive Strategies. Appl. Sci..

[39] Prieto-Lage I., Reguera-López-de-la-Osa X., Vázquez-Estévez C., Gutiérrez-Santiago A. (2025). Technical and Tactical Performance in Women’s Singles Pickleball: A Notational Analysis of Key Match Indicators. J. Funct. Morphol. Kinesiol..

[40] Armstrong C., Reid M., Beale C., Girard O. (2023). A Comparison of Match Load Between Padel and Singles and Doubles Tennis. Int. J. Sports Physiol. Perform..

[41] Silva L.M., Neiva H.P., Marques M.C., Izquierdo M., Marinho D.A. (2018). Effects of Warm-up, Post-Warm-up, and Re-Warm-up Strategies on Explosive Efforts in Team Sports: A Systematic Review. Sports Med..

[42] Kapnia A.Κ., Dallas C.N., Gerodimos V., Flouris A.D. (2023). Impact of Warm-up on Muscle Temperature and Athletic Performance. Res. Q. Exerc. Sport..

[43] Dobbs W.C., Tolusso D.V., Fedewa M.V., Esco M.R. (2019). Effect of Postactivation Potentiation on Explosive Vertical Jump: A Systematic Review and Meta-Analysis. J. Strength. Cond. Res..

[44] Seitz L.B., Haff G.G. (2016). Factors Modulating Post-Activation Potentiation of Jump, Sprint, Throw, and Upper-Body Ballistic Performances: A Systematic Review with Meta-Analysis. Sports Med..

